# Roles of basic amino acid residues in substrate binding and transport of the light-driven anion pump *Synechocystis* halorhodopsin (SyHR)

**DOI:** 10.1016/j.jbc.2025.108334

**Published:** 2025-02-19

**Authors:** Masaki Nakama, Tomoyasu Noji, Keiichi Kojima, Susumu Yoshizawa, Hiroshi Ishikita, Yuki Sudo

**Affiliations:** 1 Graduate School of Medicine, Dentistry and Pharmaceutical Sciences, Okayama University, Okayama, Japan; 2 Department of Applied Chemistry, The University of Tokyo, Bunkyo-ku, Tokyo, Japan; 3 Research Center for Advanced Science and Technology, The University of Tokyo, Meguro-ku, Tokyo, Japan; 4 Faculty of Medicine, Dentistry and Pharmaceutical Sciences, Okayama University, Okayama, Japan; 5 Atmosphere and Ocean Research Institute, The University of Tokyo, Chiba, Japan

**Keywords:** microbial rhodopsin, anion transport, retinal, membrane protein, photobiology

## Abstract

Microbial rhodopsins are photoreceptive seven-transmembrane α-helical proteins, many of which function as ion transporters, primarily for small monovalent ions such as Na^+^, K^+^, Cl^−^, Br^−^, and I^−^. *Synechocystis* halorhodopsin (SyHR), identified from the cyanobacterium *Synechocystis* sp. PCC 7509, uniquely transports the polyatomic divalent SO_4_^2−^ inward, in addition to monovalent anions (Cl^−^ and Br^−^). In this study, we conducted alanine-scanning mutagenesis on twelve basic amino acid residues to investigate the anion transport mechanism of SyHR. We quantitatively evaluated the Cl^−^ and SO_4_^2−^ transport activities of the WT SyHR and its mutants. The results showed a strong correlation between the Cl^−^ and SO_4_^2−^ transport activities among them (*R* = 0.94), suggesting a shared pathway for both anions. Notably, the R71A mutation selectively abolished SO_4_^2−^ transport activity while maintaining Cl^−^ transport, whereas the H167A mutation significantly impaired both Cl^−^ and SO_4_^2−^ transport. Furthermore, spectroscopic analysis revealed that the R71A mutant lost its ability to bind SO_4_^2−^ due to the absence of a positive charge, while the H167A mutant failed to accumulate the O intermediate during the photoreaction cycle (photocycle) due to reduced hydrophilicity. Additionally, computational analysis revealed the SO_4_^2−^ binding modes and clarified the roles of residues involved in its binding around the retinal chromophore. Based on these findings and previous structural information, we propose that the positive charge and hydrophilicity of Arg71 and His167 are crucial for the formation of the characteristic initial and transient anion-binding site of SyHR, enabling its unique ability to bind and transport both Cl^−^ and SO_4_^2−^.

Microbial rhodopsins are seven-transmembrane α-helical proteins that contain retinal (vitamin-A aldehyde) as a chromophore to absorb light (1, 2). The chromophore retinal forms a protonated Schiff base linkage with a conserved lysine residue on the seventh helix (helix G) of the protein moiety. Microbial rhodopsins are widely distributed across archaea, bacteria, eukaryotes, and viruses, exhibiting various molecular functions such as light-driven ion pumps, light-gated ion channels, phototactic sensors, and light-dependent enzymes (Fig. 1*A*) (1, 3). Upon absorbing light, microbial rhodopsins form several spectroscopically distinctive photointermediates (*e.g.*, K, L, M, N, and O intermediates), and then return to the initial unphotolyzed state (Fig. 1*B*). During this cyclic reaction, known as photocycle, structural changes in the protein moiety occur, driving the corresponding molecular functions.

**Figure 1:**
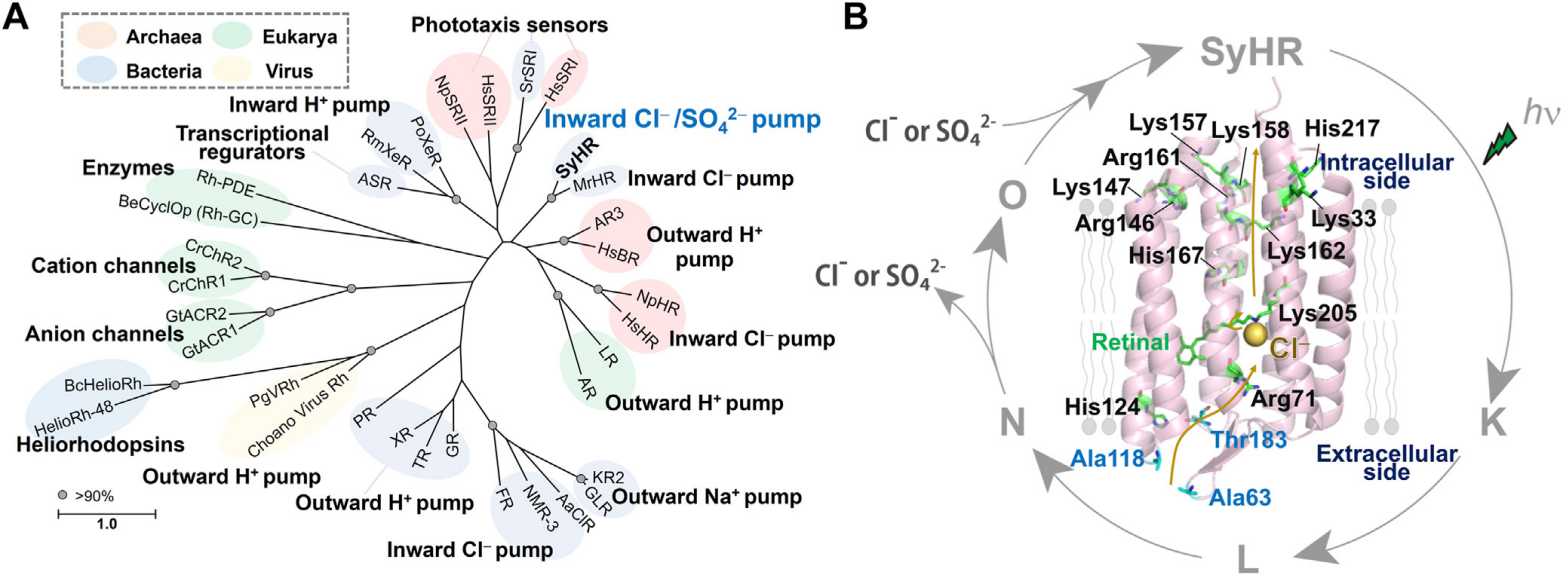
**Phylogenetic relationship of microbial rhodopsins and proposed anion transport mechanism in SyHR during the photocycle.***A*, the phylogenetic tree of known microbial rhodopsins including SyHR. *Gray circles* indicate bootstrap values above 90%. The scale bar represents the average number of amino acid substitutions per site. The tree was constructed using the maximum likelihood method in MEGA X software (https://www.megasoftware.net/; version 10.2.2). *B*, the crystal structure of SyHR (PDB: 7ZOU) and its proposed photocycle. The 12 basic amino acid residues are highlighted on the structure, along with the residues that form the extracellular concavity for the anion entrance gate. The Cl^−^ ion is represented as an *yellow dot*. PDB, Protein Data Bank; SyHR, *Synechocystis* halorhodopsin.

A light-driven proton pump rhodopsin was first identified in the archaeon *Halobacterium salinarum* and named bacteriorhodopsin (HsBR) in 1971 (4). HsBR outwardly transports protons (H^+^) to generate proton concentration gradients across the cell membrane, which are used to produce ATP. With advances in genomics, bacteriorhodopsin-like outward proton pumps have been extensively identified not only in archaea but also in bacteria, eukaryotes, and viruses (Fig. 1*A*) (1, 3, 5, 6, 7). Structural and spectroscopic analyses revealed that proton pump rhodopsins induce continuous p*K*a changes in charged amino acids (proton acceptable groups), such as key carboxylates and the protonated Schiff base. These changes, along with structural alterations during the photocycle, enable proton transport *via* the Grotthuss mechanism (8, 9). Following the discovery of HsBR, a light-driven chloride (Cl^−^) pump rhodopsin (ClR) was identified in *H*. *salinarum* and named halorhodopsin (*Halobacterium salinarum* halorhodopsin, HsHR) in 1980 (10, 11). HsHR inwardly transports halide anions such as Cl^−^ and Br^−^. The charge of the substrate ion (cation *versus* anion) and the direction of translocation (outward *versus* inward) are opposite between HsBR and HsHR. After the discovery of HsHR, advances in genomics revealed that halorhodopsin-like ClRs, such as *Natromonas pharaonis* halorhodopsin (NpHR), *Mastigocladopsis repens* halorhodopsin (MrHR), *Nonlabens marinus* rhodopsin 3 (NmR-3) and *Fulvimarina pelagi* rhodopsin (FR), are distributed across archaea and bacteria (Fig. 1*A*) (12, 13, 14, 15). These ClRs commonly transport only monovalent anions (Cl^−^, Br^−^, and I^−^) with varying ion selectivity but do not transport polyatomic divalent anions such as SO_4_^2−^. The anion transport model has been primarily proposed based on structural and spectroscopic analyses of HsHR and NpHR (1, 16, 17, 18). Unlike outward proton pump rhodopsins, where a negatively charged carboxylate counterion stabilizes the protonated Schiff base (Asp85 in HsBR), ClRs replace this residue with a neutral one, such as threonine or asparagine (*e.g.*, Thr111 in HsHR and Thr126 NpHR). This neutral residue, along with neighboring residues (*e.g.*, Arg123 and Asp252 in NpHR), forms the initial anion-binding site, with the substrate anion acting as the counterion to the protonated Schiff base in the unphotolyzed state (18). Upon light absorption, the substrate anion (Cl^−^) is translocated into the transient anion-binding site formed by Thr218 in NpHR, located on the intracellular side across the retinal during the formation of the N intermediate (18, 19, 20). The anion is then released into the intracellular side through a hydrophilic pore formed by structural changes in the sixth helix (helix F) (19, 20). Thus, the substrate monovalent anion is selectively transported through the anion-binding sites and the hydrophilic pore, moving from the extracellular to intracellular side.

In 2017, we characterized a new member of ClR-like rhodopsins, *Synechocystis* halorhodopsin (SyHR), from the cyanobacterium *Synechocystis* sp. PCC 7509 (Genome accession no. ALVU00000000) (21). SyHR is phylogenetically related to the cyanobacterial ClR such as MrHR, and contains a TSD motif (Thr74, Ser78, and Asp85) similar to that of MrHR (Fig. S1). Remarkably, SyHR inwardly transports not only monovalent ions (Cl^−^ and Br^−^) but also the divalent anion SO_4_^2−^ upon illumination, making it the first known light-driven polyatomic divalent anion transporter in nature. Our spectroscopic analysis revealed that SyHR releases and uptakes cognate anions (Cl^−^ and SO_4_^2−^) during the formation and decay of the O intermediate during the photocycle process (Fig. 1*B*). The sulfate ion SO_4_^2−^ (radius = 2.4 Å) is approximately 1.3 times larger than Cl^−^ (radius = 1.8 Å) (22), suggesting that SyHR has unique structural features in its anion-binding sites and intracellular/extracellular gates that allow it to transport SO_4_^2−^ efficiently, unlike other ClRs. Recently, the crystal structure of SyHR was solved in conditions containing Cl^−^ or SO_4_^2−^, with a resolution of 1.57 to 2.0 Å (Fig. 1*B*) (23). However, the structure of SyHR did not reveal a sulfate ion near the Schiff base in the initial anion-binding site, although a sulfate ion was detected on the protein surface. The overall structure, including the retinal binding pocket of SyHR, is similar to that of MrHR (root mean square deviation (r.m.s.d) of C_α_ = 0.359), with a small structural difference observed, such as in the intracellular cavity formed by His167 and the extracellular aqueous concave basin near Arg71 (23, 24). These structural features are thought to be important for the transport of SO_4_^2−^ in SyHR. Furthermore, several studies, including ours, have attempted to design SO_4_^2−^ transporting variants of MrHR by replacing Asn63, Pro118, and Glu182 with the corresponding residues of SyHR (Ala63, Ala118, and Thr183, respectively), which form the extracellular concavity for the anion entrance gate (25, 26). Mutational analyses suggested that the neutralizing the negative charge of Glu182 (*i.e.*, E182T mutation) and enlarging the concavity (*i.e.*, the N63A and P118A mutations) at the anion entrance gate act as crucial switches for the SO_4_^2−^ transport ability. These previous studies support the idea that the coordination of specific residues, including charged residues (*i.e.*, His, Arg, and Glu), is responsible for tuning the SO_4_^2−^ transport ability. It is also well-known that charged residues play key roles in the transport of charged ions and in determining ion selectivity in ion transporting proteins. SyHR contains twelve basic amino acid residues (*i.e.*, Lys, His, and Arg) in the α-helical domains, suggesting that these residues are involved in its Cl^−^ and SO_4_^2−^ transport abilities (Fig. 1*B*). In this study, we performed alanine-scanning mutagenesis on these twelve basic amino acid residues (*i.e.*, Lys33, Arg71, His124, Arg146, Lys147, Lys157, Lys158, Arg161, Lys162, His167, Lys205A, and His217) and analyzed possible anion binding sites employing a quantum mechanical/molecular mechanical (QM/MM) approach to investigate their roles in SyHR’s anion transport mechanism.

## Results

### Alanine-scanning mutagenesis of the twelve basic residues of SyHR

**Figure 2:**
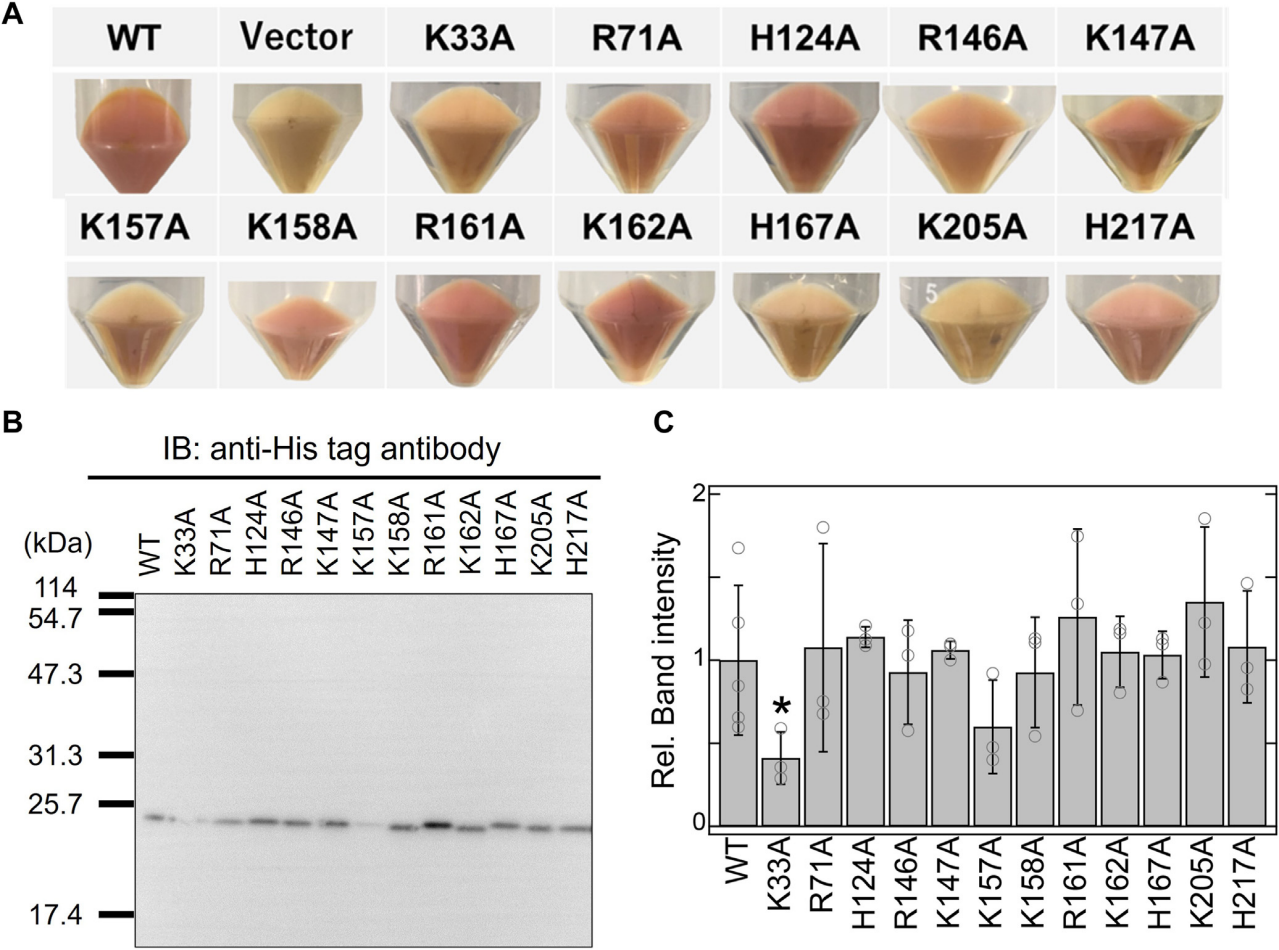
**Protein expression of the WT and mutants of SyHR in *Escherichia coli*.***A*, photographs of *E. coli* cells expressing the WT and mutants of SyHR. A *faint* or *deep red*/*orange* color was observed in all samples except for the empty vector and K205A mutant. *B*, Western blotting analysis of *E. coli* cells expressing the WT and mutants of SyHR. *C*, relative band intensities from the Western blotting with the intensity of the WT adjusted to one. All error bars represent the standard deviation (SD) from more than three independent measurements (n = 3–5). *Asterisks* (∗) indicate a significant difference from the WT SyHR (*p* < 0.05; Dunnett’s test). SyHR, *Synechocystis* halorhodopsin.

To investigate the functional roles of the twelve basic amino acid residues in SyHR, we prepared plasmids coding the single mutants in which the residues were replaced with alanine (*i.e.* K33A, R71A, H124A, R146A, K147A, K157A, K158A, R161A, K162A, H167A, K205A and H217A). Following a previous study (21), we expressed these recombinant proteins using *Escherichia coli* expression systems. The cell membranes harboring the expression plasmids for the WT SyHR and its mutants exhibited reddish/orange colors, except for the K205A mutant, suggesting that the holoproteins, as visible-light-absorbing pigments, were successfully expressed (Fig. 2*A*). Lys205 corresponds to Lys216 in HsBR, the residue responsible for retinal binding (Figs. 1*B* and S1). Thus, the absence of a reddish/orange color in the K205A mutant can be explained by the loss of retinal-binding ability. To confirm the protein expression, we performed Western blotting analysis using an anti-His tag antibody (Fig. 2*B*). An intense band was observed at approximately 25 kDa for the WT SyHR and all the mutants, including K205A. This band position closely matched the calculated molecular weight of SyHR based on its amino acid sequence (26.1 kDa), confirming successful apoprotein expression in all mutants, including the K205A mutant. The expression levels were quantitatively analyzed based on band intensities, as shown in Figure 2*C*. No significant difference in expression level was observed between the WT and the mutants, except for the K33A mutant, which exhibited an expression level approximately 2-fold lower than that of the WT.

**Figure 3:**
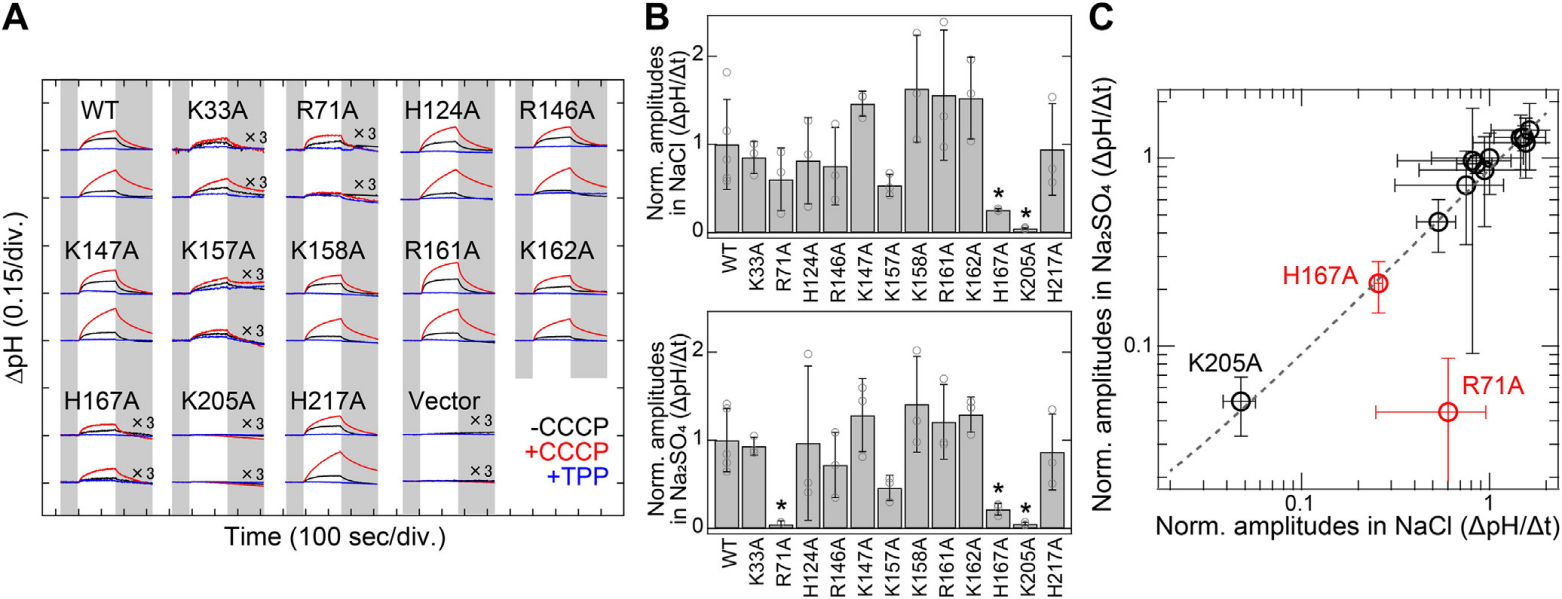
**Anion transport activity of the WT and mutants of SyHR.***A*, light-induced extracellular pH changes of *Escherichia coli* cells expressing the WT and mutants of SyHR in solutions containing 300 mM NaCl (*upper* traces) or 100 mM Na_2_SO_4_ (*lower* traces) in the absence (*black lines*) or presence (*red lines*) of CCCP, and in the presence of both CCCP and TPP^+^ (*blue lines*). *B*, quantitative evaluation of Cl^−^ and SO_4_^2−^ transport activities of the WT and mutants of SyHR. The initial slope amplitudes of the light-induced pH changes in the presence of CCCP, derived from the data in panel *A*, were normalized against the total amounts of protein (Fig. 2*C*). All error bars represent the standard deviation (SD) from more than three independent measurements (n = 3–5). *Asterisks* (∗) indicate a significant difference from the WT (*p* < 0.05; Dunnett’s test). *C*, correlation between Cl^−^ and SO_4_^2−^ transport activities of the WT and mutants of SyHR. The regression line derived from all data is depicted as a *gray dashed line*, with *R* = 0.94 (*p* < 0.01). CCCP, carbonyl cyanide *m*-chlorophenylhydrazone; SyHR, *Synechocystis* halorhodopsin; TPP^+^, tetraphenylphosphonium.

We measured light-induced extracellular pH changes in *E. coli* cells expressing the WT and mutants of SyHR to analyze their anion transport activities (Fig. 3*A*). In both NaCl and Na_2_SO_4_ solutions, light-induced pH increases were observed for the WT SyHR, and these signals were strongly enhanced by the addition of the protonophore carbonyl cyanide *m*-chlorophenylhydrazone (CCCP), as previously reported (21). These pH changes were interpreted as secondary H^+^ movements associated with the inward transport of Cl^−^ and SO_4_^2−^ by SyHR across the cell membrane. To quantitatively estimate Cl^−^ and SO_4_^2−^ transport activities, we calculated the initial slope amplitudes of extracellular pH changes from 0 to 10 s after illumination in the presence of CCCP (27). We adjusted the light intensity to 75 mW/cm^2^ for irradiation, as the relationship between light intensity and the initial slope amplitudes of pH changes exhibited a linear regression at intensities below 107 mW/cm^2^ (Fig. S2). As shown in Figure 3*A*, significant light-induced pH increases were observed for most mutants, except under certain conditions (*i.e.*, R71A in Na_2_SO_4_ solution and K205A in both NaCl and Na_2_SO_4_ solutions), albeit at varying levels. We calculated the initial slope amplitudes for the mutants with CCCP in NaCl and Na_2_SO_4_ solutions, normalizing these values by the band intensities from the Western blotting analysis to account for the total amount of protein expressed in *E. coli* cells (Fig. 3*B*). Except for the R71A mutant (red circle in Fig. 3*C*), there was a strong correlation between the normalized amplitudes in NaCl and Na_2_SO_4_ solutions for the WT SyHR and its mutants (correlation coefficient, *R* = 0.94) (Fig. 3C). This strongly suggests a common mechanism for Cl^−^ and SO_4_^2−^ transport in SyHR. Among the mutants, most (except R71A, H167A, and K205A) showed similar normalized amplitudes in both NaCl and Na_2_SO_4_ solutions, indicating the mutations (*i.e.*, K33A, H124A, R146A, K147A, K157A, K158A, R161A, K162A, and H217A) did not significantly affect the Cl^−^ and SO_4_^2−^ transport activities of SyHR (Fig. 3*B*). The normalized amplitudes of the K205A mutant in both NaCl and Na_2_SO_4_ solutions were less than 10% of those of the WT, indicating that this mutation severely impaired Cl^−^ and SO_4_^2−^ transport. This impairment is likely due to the reduced retinal binding observed in the K205A mutant, as described earlier. Notably, the normalized amplitude of the R71A mutant in Na_2_SO_4_ solution was significantly reduced (8%) compared with the WT, while in NaCl solution it was relatively comparable to the WT (65%). Additionally, the normalized amplitudes of the H167A mutant in both NaCl and Na_2_SO_4_ solutions were markedly decreased (33 and 30%, respectively) compared to the WT. These results indicate that the R71A mutation selectively abolished SO_4_^2−^ transport, while the H167A mutation impaired both Cl^−^ and SO_4_^2−^ transport in SyHR.

### Functional and spectroscopic analysis of Arg71 mutants

**Figure 4:**
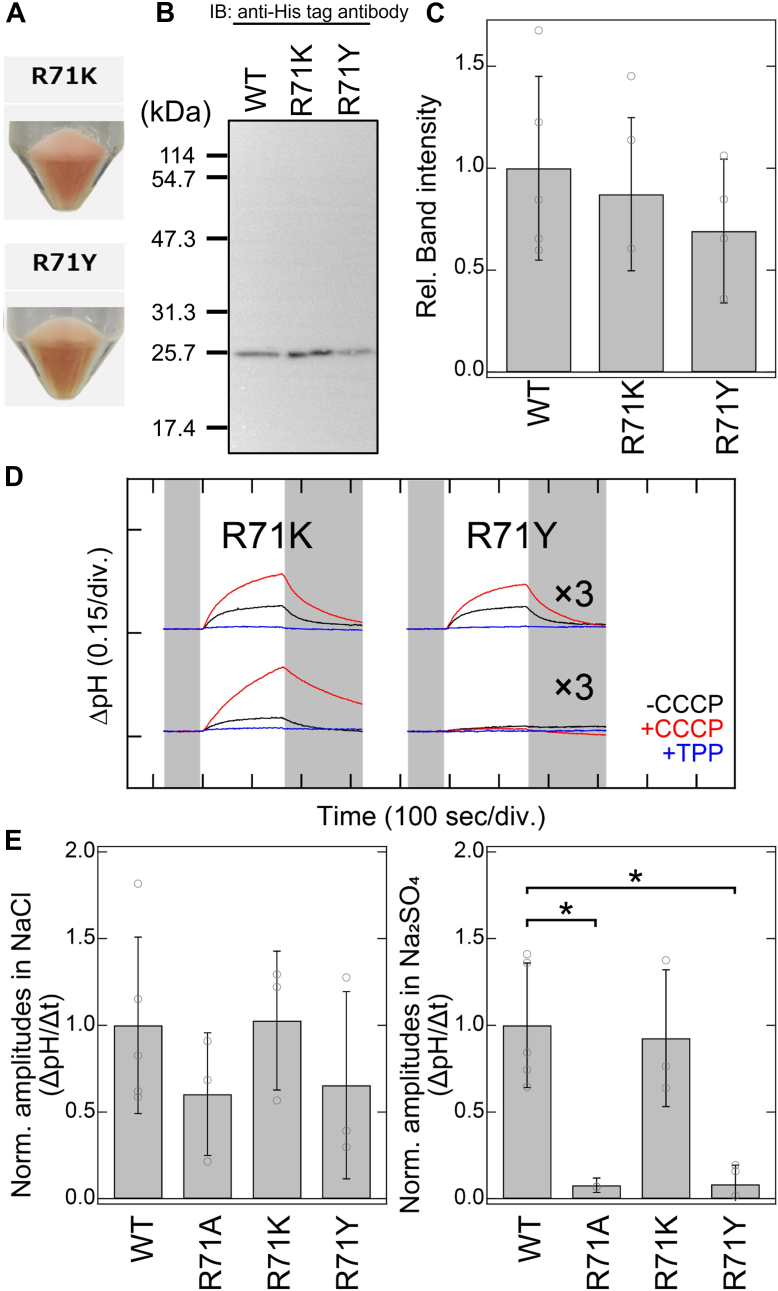
**Anion transport activities of the WT and mutants of Arg71 of SyHR.***A*, photographs of *Escherichia coli* cells expressing the R71K and R71Y mutants of SyHR. *B*, Western blotting analysis of *E. coli* cells expressing the WT, R71K, and R71Y mutants of SyHR. *C*, relative band intensities from the Western blotting with the intensity of the WT adjusted to one. *D*, light-induced extracellular pH changes of *E. coli* cells expressing the R71K and R71Y mutants of SyHR in solutions containing 300 mM NaCl (*upper* traces) or 100 mM Na_2_SO_4_ (*lower* traces) in the absence (*black lines*) or presence (*red lines*) of CCCP, and in the presence of both CCCP and TPP^+^ (*blue lines*). *E*, quantitative evaluation of Cl^−^ and SO_4_^2−^ transport activities of the WT and R71A, R71K, and R71Y mutants of SyHR. The initial slope amplitudes of the light-induced pH changes in the presence of CCCP, derived from the data in panel *D* or Figure 3, were normalized against the total amounts of protein. All error bars represent the standard deviation (SD) from more than three independent measurements (n = 3–5). *Asterisks* (∗) indicate a significant difference from the WT (*p* < 0.05; Dunnett’s test). CCCP, carbonyl cyanide *m*-chlorophenylhydrazone; SyHR, *Synechocystis* halorhodopsin; TPP^+^, tetraphenylphosphonium.

To further investigate the functional roles of Arg71, we constructed and analyzed the anion transport activities of the R71K and R71Y mutants of SyHR. Lysine, like arginine, carries a positive charge at neutral pH, whereas threonine has a similar molecular volume to arginine (the volumes for arginine and threonine are 202 Å^3^ and 204 Å^3^, respectively) (28). *E. coli* cell membranes harboring the expression plasmids for the R71K and R71Y mutants showed reddish colors similar to the WT, indicating the expression of holoproteins as visible-light absorbing pigments (Fig. 4*A*). We also measured light-induced extracellular pH changes in NaCl and Na_2_SO_4_ solutions to quantitatively obtain the normalized amplitudes of pH changes, serving as an indicator of Cl^−^ and SO_4_^2−^ transport activities (Fig. 4, *B*–*D*). The R71K mutant displayed normalized amplitudes in both NaCl and Na_2_SO_4_ solutions comparable to the WT, indicating that the Cl^−^ and SO_4_^2−^ transport activities of the mutant are similar to those of the WT (Fig. 4*E*). On the other hand, the normalized amplitude of the R71Y mutant in Na_2_SO_4_ solution was markedly reduced (8%) compared to WT, whereas the amplitude in NaCl solution remained relatively comparable (66%) to that of WT (Fig. 4*E*). The selective loss of SO_4_^2−^ transport activity in the R71Y mutant mirrors that observed in the R71A mutant, suggesting that the positive charge at position 71 is crucial for SO_4_^2−^ transport activity in SyHR.

**Figure 5:**
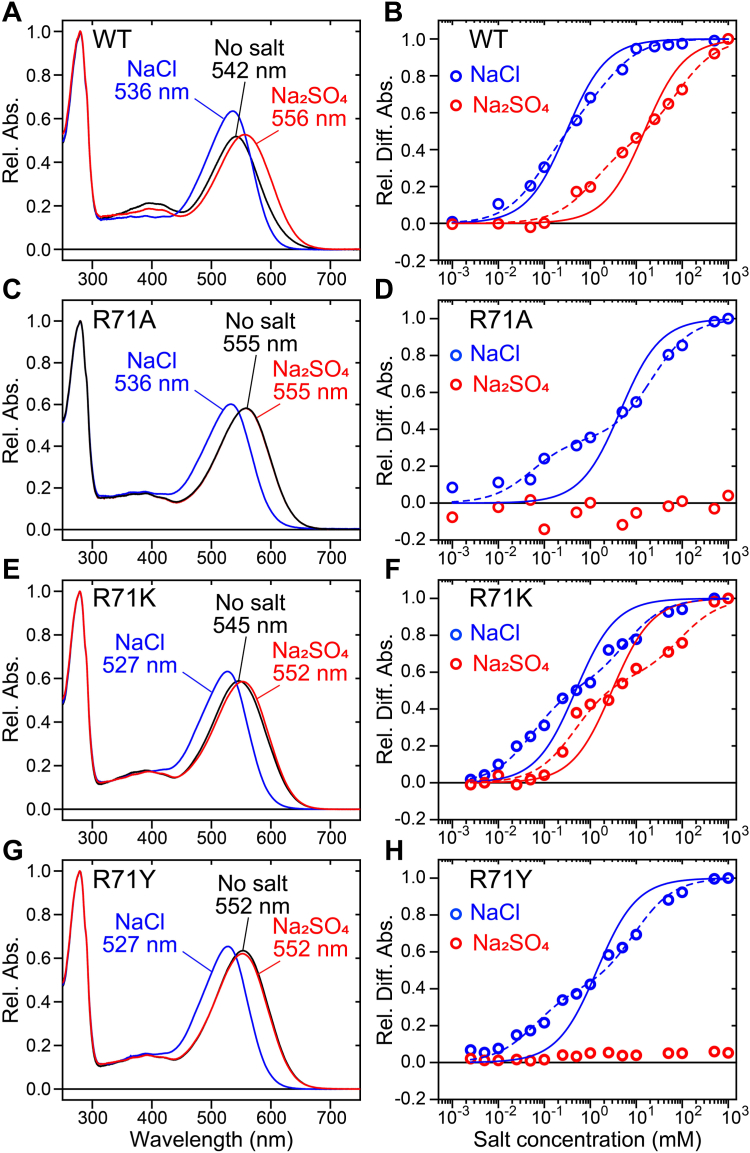
**Anion-induced spectral shifts of the WT and mutants of Arg71 of SyHR.** (*A*, *C*, *E*, and *G*) absorption spectra of the WT (*A*) and R71A (*C*), R71K (*E*), and R71Y (*G*) mutants of SyHR in the presence or absence of salts (1M NaCl or 1 M Na_2_SO_4_). (*B*, *D*, *F*, and *H*) absorption changes at the peak wavelengths in the difference spectra of the WT (*B*) and R71A (*D*), R71K (*F*), and R71Y (*H*) mutants of SyHR are plotted against the salt concentrations. The data were fitted by the linearly and double combined Hill equations (*solid* and *dashed lines*, respectively) to estimate the affinity of the anions (Table S1). SyHR, *Synechocystis* halorhodopsin.

We then measured the anion binding affinity of the R71A, R71K, and R71Y mutants for Cl^−^ and SO_4_^2−^, following our previous study (Figs. 5 and S3) (21). In this experiment, the solution pH was adjusted to approximately five to maintain the retinal Schiff base in a fully protonated state (21). The absorption spectrum of the purified WT SyHR sample exhibited blue and red shifts upon the addition of Cl^−^ and SO_4_^2−^, respectively (Fig. 5*A*). Our prior spectroscopic analysis confirmed that these spectral shifts were not due to changes in the retinal configurations (21). The difference spectra show an isosbestic point between 550 and 600 nm when adding Cl^−^ and SO_4_^2−^, indicating a two-state transition between the anion-bound and unbound states (Fig. S3). These absorbance changes were plotted against salt concentrations, and the dissociation constant (*K*_d_) was calculated using a linearly combined Hill equation (21). The *K*_d_ values of WT SyHR were estimated to be 0.59 mM for Cl^−^ and 3.6 mM for SO_4_^2−^ (Fig. 5), roughly consistent with previous estimations (0.112 and 5.81 mM) (21). Similarly, the R71K mutant showed spectral blue and red shifts upon the addition of Cl^−^ and SO_4_^2−^, respectively (Fig. 5), and its *K*_d_ values were estimated to be 0.55 mM for Cl^−^ and 3.8 mM for SO_4_^2−^, comparable to those of the WT. This indicates that the R71K mutant retains binding affinities for both anions similar to those of the WT. In contrast, the R71A and R71Y mutants did not exhibit significant spectral red shifts in the presence of SO_4_^2−^, although they showed significant spectral blue shifts upon addition of Cl^−^ (Figs. 5 and S3). The *K*_d_ values for Cl^−^ binding were estimated to be 4.5 mM for the R71A mutant and 1.4 mM for the R71Y mutant, both over twice as large as the *K*_d_ for the WT. This suggests that the R71A and R71Y mutations reduced Cl^−^ binding affinity and abolished SO_4_^2−^ binding in the initial unphotolyzed state. The loss of the SO_4_^2−^ binding ability in the R71A and R71Y mutants likely leads to the selective impairment of their SO_4_^2−^ transport activity (Fig. 4). Here, we used a linearly combined Hill equation to estimate the *K*_d_ values, assuming that a single substrate anion binds to a single binding site in SyHR protein, according to our previous study (21). However, the fitting curves show large deviations from the data points for both the WT and mutant proteins. We speculate that structural fluctuations around the anion-binding site might lead to multiple anion-binding modes with varying affinities, which could explain the observed deviations. Indeed, as described below, our theoretical analysis suggests the existence of several potential SO_4_^2−^ binding sites around Arg71. Then, we fitted the data using a double combined Hill equation to estimate the following *K*_d1_ and *K*_d2_ values: 0.094 and 2.2 mM (for Cl^−^ binding in the WT), 1.2 and 82 mM (for SO_4_^2−^ binding in the WT), 0.052 and 22 mM (for Cl^−^ binding in the R71A mutant), 0.055 and 5.9 mM (for Cl^−^ binding in the R71K mutant), 0.47 and 93 mM (for SO_4_^2−^ binding in the R71K mutant), and 0.061 and 9.0 mM (for Cl^−^ binding in the R71Y mutant) (Fig. 5 and Table S1). In either case, our results implied that the R71A and R71Y mutations abolished SO_4_^2−^ binding, suggesting the importance of the positive charge of Arg71.

### Functional and spectroscopic analysis of His167 mutants

**Figure 6:**
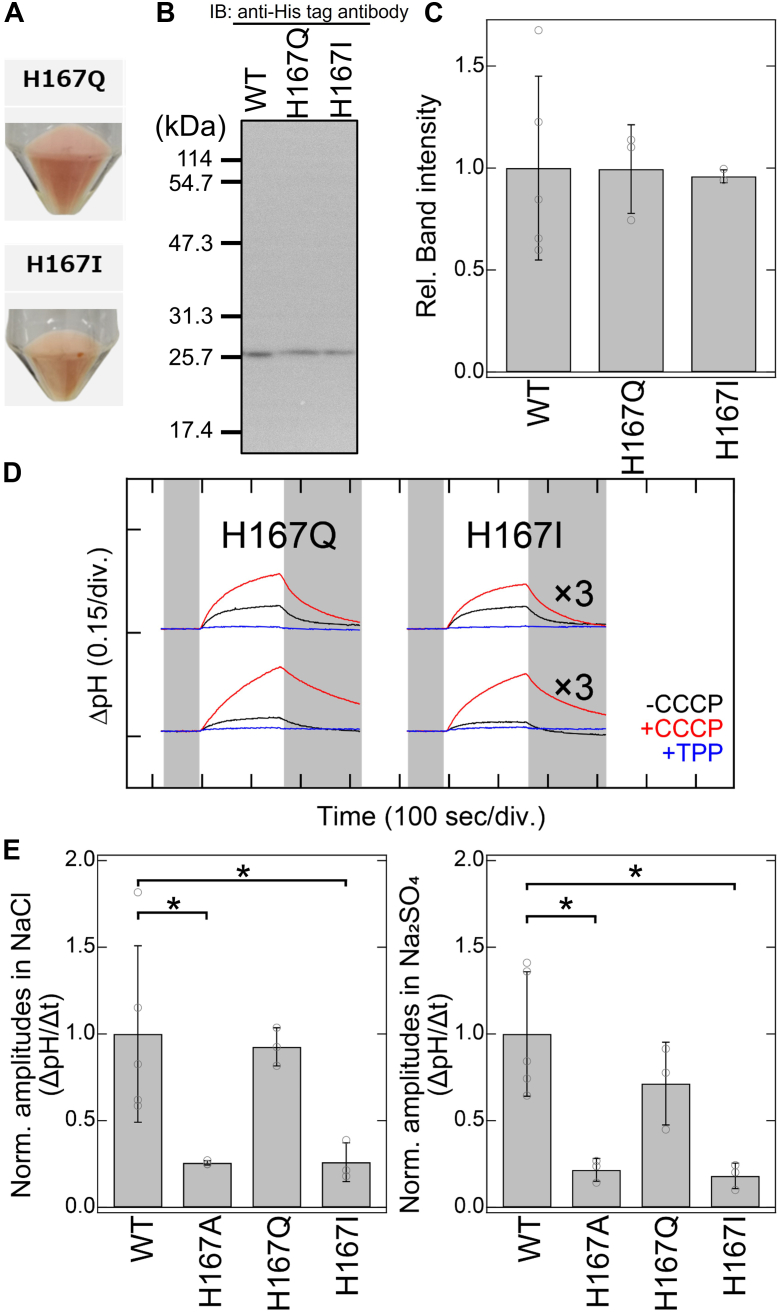
**Anion transport activities of the WT and His167 mutants of SyHR.***A*, photographs of *Escherichia coli* cells expressing the H167Q and H167I mutants of SyHR. *B*, Western blotting analysis of *E. coli* cells expressing the WT, H167Q, and H167I mutants of SyHR. *C*, relative band intensities from the Western blotting with the intensity of the WT adjusted to one. *D*, light-induced extracellular pH changes of *E. coli* cells expressing the H167Q and H167I mutants of SyHR in solutions containing 300 mM NaCl (*upper* traces) or 100 mM Na_2_SO_4_ (*lower* traces) in the absence (*black lines*) or presence (*red lines*) of CCCP, and in the presence of both CCCP and TPP^+^ (*blue lines*). *E*, quantitative evaluation of Cl^−^ and SO_4_^2−^ transport activities of the WT and H167A, H167Q, and H167I mutants of SyHR. The initial slope amplitudes of the light-induced pH changes in the presence of CCCP, derived from the data in panel *D* or Figure 3, were normalized against the total amounts of protein. All error bars represent the standard deviation (SD) from more than three independent measurements (n = 3–5). *Asterisks* (∗) indicate a significant difference from the WT (*p* < 0.05; Dunnett’s test). CCCP, carbonyl cyanide *m*-chlorophenylhydrazone; SyHR, *Synechocystis* halorhodopsin; TPP^+^, tetraphenylphosphonium.

To further investigate the functional roles of His167, we constructed and analyzed the anion transport activities of the H167Q and H167I mutants of SyHR. Glutamine has a similar polarity to histidine (polarities for histidine and glutamine are around 3.2 and 3.5, respectively), while isoleucine has a similar molecular volume to histidine (the volumes for histidine and isoleucine are 167 Å^3^ and 169 Å^3^, respectively) (28). *E. coli* cell membranes harboring expression plasmids for the H167Q and H167I mutants exhibited reddish colors similar to the WT, indicating the successful expression of holoproteins as visible-light absorbing pigments (Fig 6*A*). We also measured light-induced extracellular pH changes in NaCl and Na_2_SO_4_ solutions to quantitatively assess the normalized amplitudes of the pH changes, serving as an indicator of Cl^−^ and SO_4_^2−^ transport activities (Fig. 6, *B*–*D*). The H167Q mutant exhibited normalized amplitudes in NaCl and Na_2_SO_4_ solutions that were comparable to those of the WT, indicating similar Cl^−^ and SO_4_^2−^ transport activities. In contrast, the normalized amplitudes of the H167I mutant in NaCl and Na_2_SO_4_ solutions were significantly reduced (26 and 18%, respectively) compared to the WT. This robust impairment in both Cl^−^ and SO_4_^2−^ transport activities in the H167I mutant was also observed in the H167A mutant, suggesting that polarity at position 167 is crucial for both Cl^−^ and SO_4_^2−^ transport activities in SyHR.

**Figure 7:**
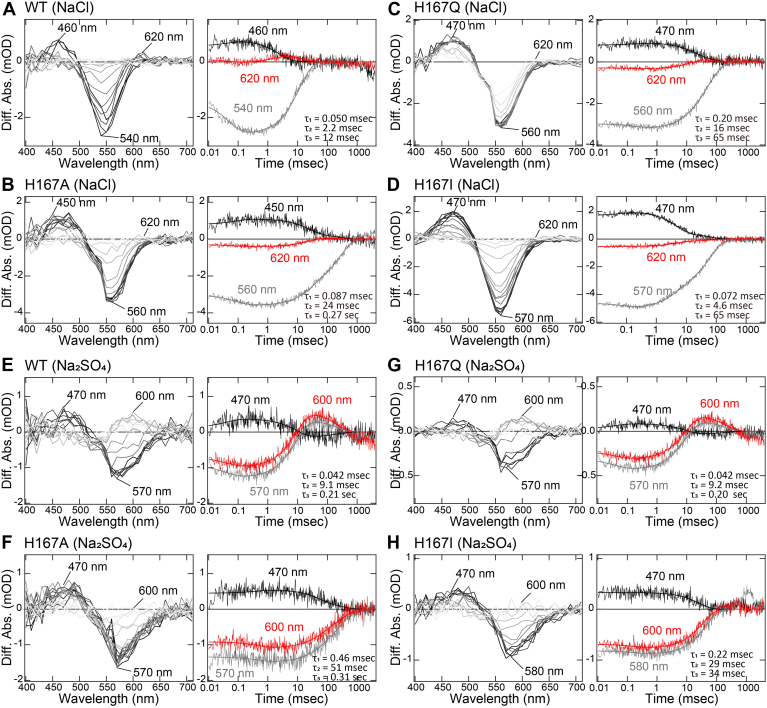
**Photoreaction kinetics of the WT and His167 mutants of SyHR.** (*A*–*D*) *Left* panel: flash-induced difference absorption spectra of the WT and H167A, H167Q, and H167I mutants of SyHR in the solution containing 300 mM NaCl over a time range of 0.01 msec to 4 s. *Right* panel: Transient absorption changes of the WT and H167A, H167Q, H167I mutants of SyHR at 470, 570, or 580, and 600 nm with fitting curves included. (*E*–*H*) *Left* panel: Flash-induced difference absorption spectra of the WT and H167A, H167Q, and H167I mutants of SyHR in the solution containing 100 mM Na_2_SO_4_ over a time range of 0.01 ms to 4 s. *Right* panel: transient absorption changes of the WT and H167A, H167Q, and H167I mutants of SyHR at 470, 570, or 580, and 600 nm with fitting curves included. SyHR, *Synechocystis* halorhodopsin.

We analyzed the anion binding affinity of the H167A mutant by examining anion-induced spectral changes to determine whether the loss of anion binding ability correlates with reduced anion transport activities (Fig. S3). Similar to the WT, the H167A mutant exhibited spectral blue and red shifts upon the addition of Cl^−^ and SO_4_^2−^, respectively, indicating that the mutant retained its anion binding abilities. The *K*_d_ values of the H167A mutant were estimated to be 0.36 mM for Cl^−^ and 6.5 mM for SO_4_^2−^, which were comparable to those of the WT (0.59 mM for Cl^−^ and 3.6 mM for SO_4_^2−^). This suggests that the anion binding ability of the H167A mutant is not significantly altered and, therefore, would not account for its reduced anion transport activities. Next, we analyzed the photocycle of the His167 mutants, as microbial rhodopsins transport ions *via* conformational changes that occur during the photocycle. Our previous study, conducted with purified SyHR sample in detergent micelles, demonstrated that the WT SyHR sequentially forms the K, L, and N/O intermediates before returning to the initial unphotolyzed state (21). Additionally, we proposed that SyHR releases and uptakes substrate anions (Cl^−^ and SO_4_^2−^) during the formation and decay of the O intermediate in the photocycle process. In this study, we performed millisecond time-resolved spectroscopic analysis of the WT SyHR and its mutants embedded in *E. coli* membranes, and assigned the photointermediates according to our previous study (Fig. 7) (21). In NaCl solution, the WT SyHR initially forms a photointermediate around 460 nm, known as the L intermediate, due to the decay of the K intermediate upon illumination (Fig. 7*A*). The L intermediate then decays, accompanied by the formation of another photointermediate around 620 nm, identified as the O intermediate. Finally, the O intermediate decays, leading to the recovery of the initial state. We plotted the absorbance changes at 540, 460, and 620 nm, corresponding to the initial state, L intermediate and O intermediate, respectively, and performed global fitting analysis to obtain the decay time constants for the K, L, and O intermediates, which were 0.050, 2.2, and 12 msec, respectively (Fig. 7*A*). We then conducted similar experiments for the H167A, H167Q, and H167I mutants of SyHR (Fig. 7, *B*–*D*). The difference absorption spectra of the H167Q mutant showed a negative peak around 560 nm due to the depletion of the initial state, and positive peaks around 470 and 620 nm due to the formation of the L and O intermediates, respectively, consistent with the WT (Fig. 7*C*). The decay time constants for the K, L, and O intermediates were 0.20, 16, and 65 msec, respectively, in the H167Q mutant. On the other hand, the difference absorption spectra of the H167A or H167I mutants showed a negative peak around 560 or 570 nm due to the depletion of the initial state and a positive peak around 450 or 470 nm due to the formation of the L intermediate, respectively (Fig. 7, *B* and *D*). Notably, a significant peak around 620 nm was not observed in the H167A and H167I mutants. Thus, these two mutants did not significantly accumulate the O intermediate, implying the loss of O intermediate formation during the photocycle process.

Furthermore, we performed time-resolved spectroscopic analysis of the WT and mutants of SyHR in Na_2_SO_4_ solution (Fig. 7, *E*–*H*). The difference absorption spectra of the WT and H167Q mutant of SyHR showed a negative peak around 570 nm due to the depletion of the initial state and positive peaks around 470 and 600 nm, corresponding to the formation of the L and O intermediates, respectively, similar to what was observed in NaCl solution (Fig. 7, *E* and *G*). Thus, the WT and H167Q mutant of SyHR sequentially form the L and O intermediates and finally return to the initial state. On the other hand, a significant peak around 600 nm was not observed in the H167A or H167I mutants, indicating that these two mutants did not significantly accumulate the O intermediate in Na_2_SO_4_ solution, as was the case in NaCl solution (Fig. 7, *F* and *H*). This suggests the loss of the O intermediate formation during the photocycle process in both NaCl and Na_2_SO_4_ solutions. Since the O intermediate plays a crucial role in the release and uptake of substrate anions during the photocycle process (21), the absence of O intermediate formation in the H167A or H167I mutants likely reflects an inhibition of efficient substrate anion movement along the intramolecular anion transport pathway of SyHR, ultimately impairing their Cl^−^ and SO_4_^2−^ transport activities.

### Structural analysis of the SO_4_^2−^ binding site

**Figure 8:**
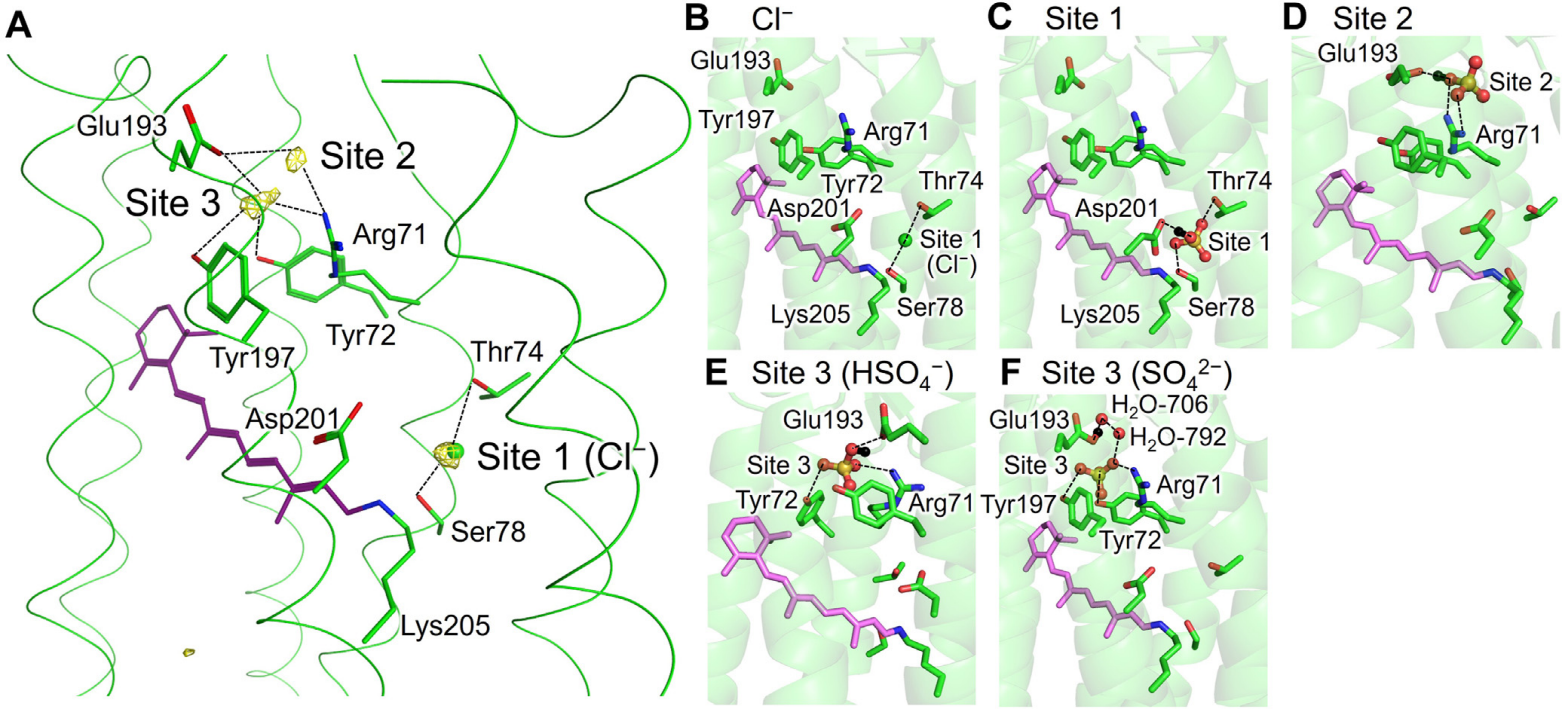
**Potential anion-binding sites near the retinal Schiff base in the SyHR structure.***A*, *yellow meshes* represent the identified anion-binding sites. The *green sphere* indicates the Cl^−^ ion identified in the original SyHR structure. *Dotted lines* indicate likely interactions in each binding site. *B*–*F*, detailed views of anion-binding sites. (*B*) Cl^−^. *C*, site 1. *D*, site 2. *E*, site 3 (HSO_4_^−^). and *F*, site 3 (SO_4_^2−^). *Black spheres* indicate hydrogen atoms involved in interactions between the anions and H-bond partner residues. *Dotted lines* indicate representative H-bonds. SyHR, *Synechocystis* halorhodopsin.

Using a three-dimensional reference interaction site model coupled with Placevent analysis (29), three potential anion binding sites were identified in the SyHR structure (Fig. 8). These sites are as follows:

Site 1: The original Cl^−^ binding site formed by Thr74, Ser78, and Asp201.

Site 2: A site formed by Arg71 and Glu193.

Site 3: A site formed by Arg71, Tyr72, Tyr197, and water molecules forming hydrogen bonds (H-bonds) with Glu193.

In water, the p*K*_a_ value for the first dissociation of H_2_SO_4_, p*K*_a_ (H_2_SO_4_/HSO_4_^−^), is extremely low, whereas the p*K*_a_ value for the second dissociation, p*K*_a_ (HSO_4_^−^/SO_4_^2−^), is elevated to approximately 2. These p*K*_a_ values suggest that the anion at the candidate binding sites in the SyHR structure is likely to exist as either HSO_4_^−^ or SO_4_^2−^. The protonation patterns of titratable residues in SyHR were analyzed for both HSO_4_^−^ and SO_4_^2−^ at each candidate binding site.

Site 1: The protonation pattern calculated by solving the linear Poisson-Boltzmann equation indicates that, if SO_4_^2−^ is assumed to bind at Site 1, Asp201 becomes protonated. However, in subsequent QM/MM calculations, proton transfer occurs from protonated Asp201 to SO_4_^2−^, resulting in the formation of HSO_4_^−^ and ionized Asp201 (Table 1). Therefore, HSO_4_^−^, not SO_4_^2−^, is stable at site 1, with Asp201 acting as the H-bond acceptor. Site 2: Similarly, if SO_4_^2−^ is assumed to bind at site 2, the calculated protonation pattern indicates that Glu193 becomes protonated. However, in subsequent QM/MM calculations, proton transfer occurs from protonated Glu193 to SO_4_^2−^, leading to the formation of HSO_4_^−^ and ionized Glu193 (Table 1). Consequently, HSO_4_^−^, not SO_4_^2−^, is stable at site 2, with Glu193 serving as the H-bond acceptor. Site 3: site 3 shares structural similarities with site 2 due to their proximity but differs in that Glu193 indirectly forms an H-bond with this site *via* a water molecule (Fig. 8, *E* and *F*). As a result, both SO_4_^2−^ and HSO_4_^−^ are energetically stable at site 3, with no proton transfer observed in QM/MM calculations. When HSO_4_^−^ is present, the adjacent water molecule accepts an H-bond from HSO_4_^−^, and Glu193 becomes ionized. When SO_4_^2−^ is present, the adjacent water molecule donates an H-bond to SO_4_^2−^, and Glu193 remains protonated.

**Table 1:** Protonation patterns initially calculated by solving the linear Poisson-Boltzmann equation and subsequently evaluated using QM/MM calculations

Site	Protonation pattern			QM/MM		
	Anion	Glu193	Asp201	Anion	Glu193	Asp201
Site 1	HSO_4_^−^	deprot.	deprot.	HSO_4_^−^	deprot.	deprot.
	SO_4_^2−^	deprot.	prot.			
Site 2	HSO_4_^−^	deprot.	deprot.	HSO_4_^−^	deprot.	deprot.
	SO_4_^2−^	deprot.	prot.			
Site 3	HSO_4_^−^	deprot.	deprot.	HSO_4_^−^	deprot.	deprot.
	SO_4_^2−^	prot.	deprot.	SO_4_^2−^	prot.	deprot.

Deprot, deprotonated; Prot, protonated.

These results suggest that HSO_4_^−^ rather than SO_4_^2−^ is more likely to exist near the retinal Schiff base in SyHR. While it is energetically possible for SO_4_^2−^ to exist at the binding site (site 3), this scenario would require a process in which SO_4_^2−^ reaches the site without interacting with acidic residues, such as Glu193 and Asp201, along the pathway.

## Discussion

In this study, we performed a mutational analysis of the twelve basic amino acid residues in the Cl^−^/SO_4_^2−^ transporting rhodopsin SyHR, revealing a strong correlation between Cl^−^ and SO_4_^2−^ transport activity (*R* = 0.94) (Fig. 3*C*). These findings strongly suggest a shared anion transport pathway for Cl^−^ and SO_4_^2−^. Given that these two anions (Cl^−^ and SO_4_^2−^) carry one and two negative charges, respectively, under our experimental conditions (pH approximately 5.0–7.0), an additional charge compensation mechanism is required for SO_4_^2−^ transport. We propose two possible mechanisms for this compensation: (i) despite its low p*K*_a_ (approximately 1.9), sulfate ions predominantly exist in their monovalent form (HSO_4_^−^) due to interactions with the protein moiety, or (ii) positively charged residues such as Arg, Lys, and His, along with water molecules, compensate the negative charge of SO_4_^2−^. We aim to further investigate these hypotheses through additional biochemical and biophysical experiments in the near future.

Based on our mutational analysis, we identified several structurally and functionally important residues in SyHR including Lys33, Arg71, His167, and Lys205. Here, we discuss their proposed roles in relation to the protein’s structure and function. The K205A mutant expressed apoprotein, but both its reddish/orange color and anion transport activity were lost (Figs. 2 and 3). Lys205 is conserved among microbial rhodopsins such as HsBR and NpHR, where it functions as a retinal binding residue that forms a protonated Schiff base linkage (Fig. S1). Therefore, we conclude that Lys205 in SyHR serves as the retinal-binding residue. Regarding Lys33, the expression level of the K33A mutant was significantly lower than that of the WT (Fig. 2). In addition, the expression level of the K157A mutant appears to be lower than that of the WT, although the difference is not statistically significant. In the crystal structure, Lys33 and Lys157 are located on the surface of the protein (Fig. 1), suggesting their potential roles in interacting with negatively charged phospholipids on the membrane surface. We speculate that substituting Lys33 and Lys157 with the neutral residue alanine disrupts the electrostatic interaction, leading to destabilization of the recombinant protein and reduced expression levels in *E. coli* cells.

Regarding the role of Arg71, the R71A, and R71Y mutations selectively abolished the SO_4_^2−^ transport activity, while the R71K mutation maintained transport activities for both Cl^−^ and SO_4_^2−^, similar to the WT (Fig. 4). Furthermore, the R71A and R71Y mutations reduced or abolished Cl^−^ and SO_4_^2−^ binding abilities, respectively, whereas the R71K mutation retained binding abilities comparable to the WT (Fig. 5). These results suggest that the positive charge at position 71 is crucial for maintaining the binding abilities for Cl^−^ and SO_4_^2−^ in the unphotolyzed state. The crystal structure of SyHR revealed that a Cl^−^ ion located in the initial anion-binding site near the Schiff base and Arg71 (site 1 in Fig. 8, *A* and *B*) (23). While our theoretical analysis suggests that SO_4_^2−^ (or HSO_4_^−^) can occupy the original Cl^−^ binding site (site 1), it also occupy additional sites (sites 2 and 3) near Arg71. Considering that the direction of the anion-induced spectral shift differs between Cl^−^ and SO_4_^2−^ (blue-shift versus red-shift, respectively) (Fig. 5) (21), we propose that the initial sulfate-binding site is either site 2 or 3. These suggest that the positive charge of Arg71 contributes to the stable localization of substrate Cl^−^ and SO_4_^2−^ through electrostatic interactions. On the other hand, substituting Arg71 with the neutral residues, as in the R71A and R71Y mutations, would disrupt this electrostatic interaction and alter the local structure of the binding site, resulting in decreased Cl^−^ and SO_4_^2−^ binding affinities. Since SO_4_^2−^ binding affinity is six times lower than that of Cl^−^ in the WT SyHR, this reduction leads to the complete loss of SO_4_^2−^ binding ability in the R71A and R71Y mutants. While Arg71 plays a key role in SO_4_^2−^ binding in SyHR, it is also widely conserved among ClRs, forming part of the initial anion-binding site in these proteins (Fig. S1). However, Glu193, which forms part of the initial sulfate-binding sites (sites 2 and 3), is not conserved in typical ClRs (*e.g.*, HsHR and NpHR), suggesting that structural differences contribute to SyHR’s unique ion selectivity. Notably, MrHR also possesses the corresponding Glu residue (Glu192 in MrHR), consistent with the observation that MrHR mutants exhibit SO_4_^2−^ transport activity (25, 26). We speculate that the characteristic initial sulfate-binding site in SyHR, including the positive charge of Arg71 and Glu193, enables its distinctive ability to bind SO_4_^2−^ (Fig. 8*A*).

Regarding the role of His167, the H167A, and H167I mutations significantly decreased Cl^−^ and SO_4_^2−^ transport activities, while the H167Q mutation maintained similar transport activities compared to the WT (Fig. 6). Furthermore, the H167A and H167I mutations reduced the accumulation of the O intermediate during the photocycle, whereas the H167Q mutation preserved a comparable level of O intermediate accumulation to that of the WT (Fig. 7). These results suggest that the polarity at position 167 is crucial for the formation of the O intermediate, which is essential for efficient Cl^−^ and SO_4_^2−^ transport in SyHR. The crystal structure of SyHR revealed that His167, Leu170, Typ171, and Ser204 form the intracellular cavity (Fig. S5) (23). This corresponding intracellular cavity is known to function as a transient anion-binding site, which is critical for anion release to the intracellular side during the decay process of the O intermediate in NpHR (19, 20). Therefore, it is proposed that the polarity of H167 facilitates the localization of substrate Cl^−^ and SO_4_^2−^ within the transient anion-binding site by increasing the hydrophilicity in that area. On the other hand, substituting His167 with alanine or isoleucine, both of which have lower polarity, would decrease the hydrophilicity surrounding the transient anion-binding site. This change could inhibit the transition of Cl^−^ and SO_4_^2−^ from the initial anion-binding site to the transient anion-binding site beyond the retinal Schiff base. The inhibition of this transition would lead to the loss of O intermediate formation, as the O intermediate is generated in conjugation with the release of substrate Cl^−^ and SO_4_^2−^ from the transient anion-binding site to the intracellular side. In contrast to SyHR, HsHR, and NpHR have threonine (Thr203 and Thr218, respectively) in place of His167 (Fig. S5). Given that the threonine has lower polarity than histidine, we speculate that the characteristic high hydrophilicity around the transient anion-binding site in SyHR contributes to its ability to transport SO_4_^2−^ in addition to Cl^−^. It should be noted that the extracellular pH changes of *E. coli* in the presence of CCCP were measured as an indicator of Cl^−^ and SO_4_^2−^ transport activities, rather than through direct observation of substrate ion transport. In the future, it will be necessary to validate our estimation of Cl^−^ and SO_4_^2−^ transport activities by performing direct transport measurements using purified WT and mutant SyHR proteins reconstituted into proteoliposomes.

**Figure 9:**
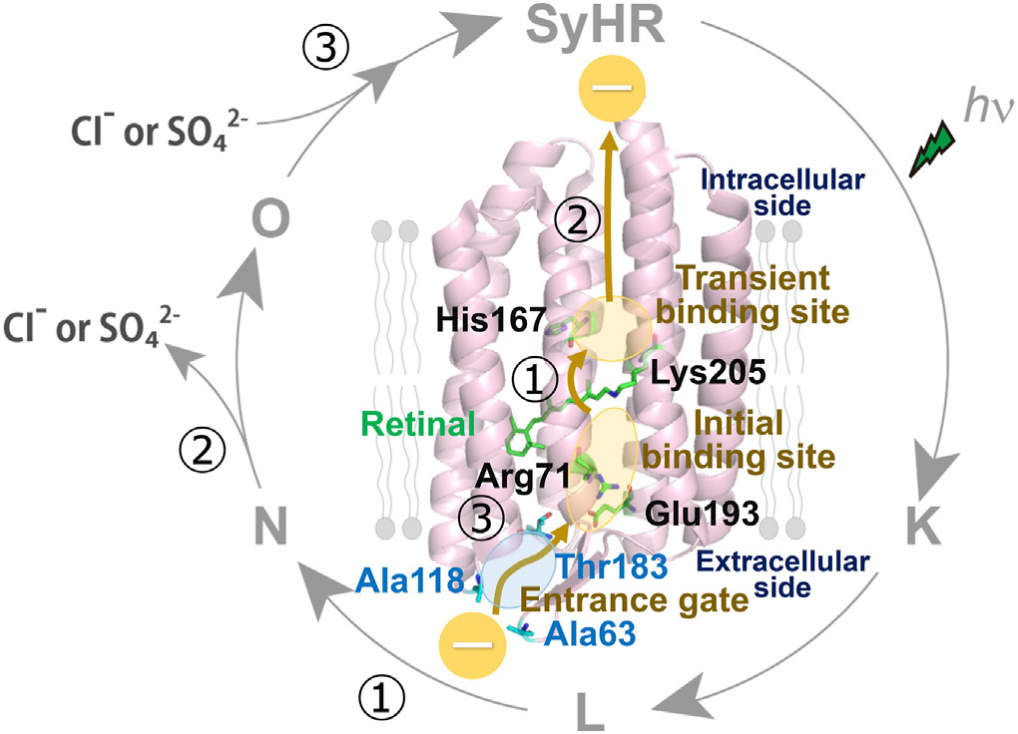
**A hypothetical anion transport model of SyHR.** An inward anion transport mechanism in SyHR during the photocycle. The proposed pathway for anion transport and key residues are marked on the structure of SyHR (PDB: 7ZOU). PDB, Protein Data Bank; SyHR, *Synechocystis* halorhodopsin.

Based on our results and previous spectroscopic and structural studies (21, 23, 25, 26), we propose a putative anion transport model for SyHR (Fig. 9). In the initial unphotolyzed state, the substrate anion (Cl^−^ or SO_4_^2−^/HSO_4_^−^) is located in the different initial anion-binding sites near the Schiff base, where the positive charge of Arg71 facilitates robust anion binding through electrostatic interactions. Upon light absorption, *trans*-*cis* isomerization of the retinal occurs, leading to the formation of the L intermediate *via* the K intermediate. During the transition from the L to N intermediate, the substrate anion is transferred from the initial anion-binding site to the transient anion-binding site, where the high hydrophilicity conferred by His167 promotes strong anion binding. Subsequently, during the transition from the N to O intermediate, the substrate anion is released from the transition anion-binding site to the intracellular side. Finally, during the transition from the O intermediate back to the initial state, the substrate anion is taken up from the extracellular anion entrance gate, which comprises Ala63, Ala118, and Thr183. In addition, several basic amino acid residues, such as Arg59 and Arg65, are located around the gate (Fig. S1). Furthermore, functional and spectroscopic experiments will be necessary to elucidate their roles in the anion uptake mechanism of SyHR. Consequently, the substrate anion is transported from the extracellular side to the intracellular side during a single photocycle.

## Conclusion

We conducted a mutational analysis of twelve basic amino acid residues in the Cl^−^/SO_4_^2−^ transporting rhodopsin SyHR. Our results strongly suggest a shared anion transport pathway for Cl^−^ and SO_4_^2−^. Specifically, Arg71 and His167 play critical roles in the formation of the characteristic initial and transient anion-binding site of SyHR, facilitating its unique binding and transport abilities for these anions. Our proposed anion transport mechanism provides valuable insights for the rational design of new types of rhodopsins with the capability to transport alternative anions.

## Experimental procedures

### Gene preparation, protein expression, and anion transport measurements

The complementary DNA for SyHR (Genbank accession number: WP_009632765) was optimized for codon usage in *E. coli* cells and fused to a C-terminal sequence encoding a hexahistidine tag (His tag) (21). The fusion product was then inserted into the pET21a plasmid vector (Novagen), as previously described (21, 30). Mutations in the SyHR complementary DNA were constructed using the In-Fusion Cloning Kit (Takara Bio) or the SLiCE method (27, 31). Recombinant proteins were expressed in *E. coli* BL21 (DE3) as previously described (21). *E*. *coli* cells harboring the expression plasmid were incubated in 5 ml LB medium containing 100 μg/ml ampicillin and grown at 37 °C for 15 h. After the preculture, the growth medium was transferred directly to 100 ml LB medium containing 50 μg/ml ampicillin and grown at 37 °C until the absorbance at 660 nm reached 1.5 to 1.7. Then, IPTG was added to a final concentration of 1.0 mM, along with all-*trans* retinal at a final concentration of 10 μM, and the culture was incubated at 37 °C for an additional 3 h to induce protein expression. The cells were then collected by centrifugation at 6500*g* for 10 min at 4 °C.

Anion transport activity was measured by light-induced pH changes using essentially a method similar to that previously described (21). *E. coli* cells were suspended and washed three times in a solution containing 300 mM NaCl or 100 mM Na_2_SO_4_ to remove the LB culture medium. After washing, the cells were resuspended in the same solutions, and the absorbance at 660 nm of all samples was set at 8. The samples were kept in the dark and then illuminated with a Xenon lamp (Asahi Spectra Co Ltd, Japan) through a 440 nm long-pass filter (>420 nm). The light intensity was measured and adjusted to approximately 75 mW/cm^2^ using an optical power meter (Hioki) equipped with an optical sensor (Hioki). When necessary, the protonophore carbonyl cyanide *m*-chlorophenylhydrazone (CCCP, Sigma-Aldrich) or tetraphenylphosphonium ion (TPP^+^) was added to the medium at final concentrations of 10 μM or 30 mM, respectively. The temperature of the samples was maintained at 25 °C using a thermostat. The initial slope amplitudes of the light-induced pH changes from 0 to 10 s after light irradiation, in the presence of CCCP, were used as the measure of anion transport activity for the WT and mutants of SyHR, according to previous studies (27, 32).

### Western blotting analysis

Western blotting analysis, followed by SDS-PAGE, was performed according to standard methods as previously described (27, 33). *E. coli* cells were suspended in a buffer containing 20 mM Tris–HCl (pH 8.0) and 100 mM NaCl. The absorbance at 660 nm of all samples was initially set at 8, and the cell suspensions were then diluted 50-fold with the same buffer. Diluted samples (5 μl) were mixed with SDS-PAGE loading buffer (5 μl) containing 5% 2-mercaptoethanol and heated at 95 ˚C for 5 min. The samples (10 μl) were then separated by 12% acrylamide SDS-PAGE. Immunoblotting analysis was conducted using an anti-His tag HRP conjugate antibody (Medical & Biological Laboratories, Co., Ltd) according to the manufacturer’s instructions. To assess the protein expression levels of the WT and mutants of SyHR, band intensities were quantitatively evaluated using ImageJ software (https://imagej.net/ij/index.html; NIH, USA).

### Protein purification and UV-visible spectroscopy

Recombinant proteins were expressed in *E. coli* BL21 (DE3) and purified using affinity column chromatography, as previously described (21, 34). *E. coli* cells expressing the WT and mutants of SyHR were suspended in 50 mM Tris–HCl (pH 8.0) buffer containing 300 mM NaCl and then disrupted by sonication in an ice-cold water bath. The crude membrane fraction was collected *via* ultracentrifugation and subsequently solubilized at 4 °C with 1.5% (w/v) n-dodecyl-β-D-maltoside (DDM) in 50 mM Tris–HCl (pH 8.0) buffer containing 300 mM NaCl. After a second ultracentrifugation, the supernatant was applied to a HisTrap FF Ni^2+^-NTA affinity chromatography column (Cytiva). The Ni^2+^-NTA resin in the column was thoroughly washed with 50 mM Tris–HCl (pH 8.0) buffer containing 1 M NaCl, 0.1% (w/v) DDM, and 20 mM imidazole. The SyHR protein was then eluted by increasing the imidazole concentration to 1 M. Finally, the purified SyHR was concentrated and exchanged into the appropriate buffer solution by centrifugation using an Amicon Ultra filter. For the anion titration experiment, the purified proteins were suspended in 10 mM Mops buffer containing 0.05% (w/v) DDM. The absorption spectra of the WT and mutants of SyHR were measured by adding NaCl or Na_2_SO_4_ at concentrations ranging from 0 to 1 M, using a UV-2450 spectrophotometer (Shimadzu) at room temperature (approximately 23–28 °C). The anion-induced absorbance changes at the peak wavelengths were plotted against the logarithm of the anion concentration and analyzed by fitting the data to the Hill equation (Equations 1 and 2), as described in previous studies (21).

(Eq. 1) $$\Delta A=V_{\max}\times \frac{x}{K_{d}+x}$$

(Eq. 2) $$\Delta A=V_{\max\;1}\times \frac{x}{K_{d1}+x}+V_{\max\;2}\times \frac{x}{K_{d2}+x}$$

Here, *ΔA* is the absorption change; *V*_*max*_, *V*_*max1*_, and *V*_*max2*_ are the maximum absorption changes; *K*_*d*_, *K*_*d1*_, and *K*_*d2*_ are the dissociation constants; *x* is the anion concentration. The data were fitted using the Hill equations (Equations 1 and 2), assuming that *V*_*max*_ and the sum of *V*_*max1*_ and *V*_*max2*_ are fixed at 1.

### Time-resolved spectroscopic analysis

Transient time-resolved spectroscopic analysis was conducted using *E. coli* membranes (35). Crude membrane fractions expressing the WT and mutants of SyHR were prepared as described in previous studies (32, 34, 35) and suspended in 10 mM Mops (pH 6.5) buffer containing either 300 mM NaCl or 100 mM Na_2_SO_4_. Absorption spectra were recorded from 420 to 710 nm at 10 nm intervals over a time range of 0.01 msec to 4 s, utilizing a homemade computer-controlled flash-photolysis system equipped with an Nd:YAG laser (Surelite I-10, Continuum) as the actinic light source (27, 34). The wavelength of the actinic pulse was set to 535 or 545 nm (4 nsec) using an optical parametric oscillator (Surelite OPO Plus, Continuum), with the pulse intensity adjusted to 2 mJ/pulse. To enhance signal-to-noise ratio, the results of 400 to 800 traces were averaged at each wavelength. The samples were maintained at 25 °C using a cell holder equipped with a temperature-controlled circulating water bath. Data analysis involved an irreversible sequential model, as reported previously (34), with the transient absorption changes at the selected wavelengths simultaneously fitted using a triple-exponential function.

### Protonation pattern of SyHR

The atomic coordinates were obtained from the X-ray structure of SyHR (Protein Data Bank code, 7ZOU). During the optimization process for hydrogen atom positions using CHARMM (36), all heavy atom positions were kept fixed. All titratable groups (*e.g.*, acidic and basic groups) were ionized, and the retinal Schiff base was protonated. Atomic partial charges for amino acids and the protonated retinal Schiff base were obtained from the CHARMM22 parameter set (37). The protonation pattern of the titratable residues was calculated by solving the linear Poisson-Boltzmann equation using the MEAD program (38). All computations were performed at 300 K, pH 7.0, and an ionic strength of 100 mM. For p*K*_a_ calculations, crystal water molecules were removed, and water molecules were implicitly considered using a dielectric constant of 80. To determine the protonation pattern of titratable sites in the protein, the calculated p*K*_a_ difference between the protein site and the reference system was added to the known reference p*K*_a_ value. The experimentally measured p*K*_a_ values used as references were 12.0 for Arg, 4.0 for Asp, 9.5 for Cys, 4.4 for Glu, 10.4 for Lys, 9.6 for Tyr (39), and 7.0 and 6.6 for the N_ε_ and N_δ_ sites of His, respectively (40, 41, 42). Protonation patterns were sampled using a Monte Carlo method with Karlsberg (43). The linear Poisson-Boltzmann equation was solved through a three-step grid-focusing procedure at resolutions of 2.5 Å, 1.0 Å, and 0.3 Å. Monte Carlo sampling provided the probabilities ([protonated] and [deprotonated]) for the two protonation states. The resulting protonation patterns were subsequently used in QM/MM calculations.

### Modeling the SO_4_^2−^ binding structure

To model the SO_4_^2−^ binding structure, candidate SO_4_^2−^ binding sites near the retinal Schiff base were initially identified as a representative anion (Cl^−^) binding site. These candidate sites were determined in the Cl^−^removed structure using three-dimensional reference interaction site model coupled with Placevent analysis (29). For site 1, the Cl^−^ ion in the crystal structure was replaced with HSO_4_^−^ or SO_4_^2−^. For sites 2 and 3, the water molecule nearest to the identified binding site was replaced with HSO_4_^−^ or SO_4_^2−^. To prevent steric hindrance, the nearest water molecule to SO_4_^2−^ was removed. The resulting anion-bound structure was then optimized using a QM/MM approach. The restricted density functional theory method was employed with the B3LYP functional and LACVP∗ basis sets, using the QSite (44) program. The QM region included the retinal Schiff base (including the Lys205 side chain); side chains of Tyr50, Arg71, Tyr72, Thr74, Trp75, Ser78, Tyr174, Glu193, Tyr197, and Asp201; as well as water molecules located near the Schiff base (H_2_O-706, 710, 717, 727, 729, 740, 742, 748, 768, 784, 787, 792, 795, and 816). All other protein components and water molecules in the SyHR crystal structure were included in the MM region. Atomic coordinates were fully relaxed in the QM region. In the MM region, the positions of H atoms were optimized using the OPLS2005 force field (45), while the positions of heavy atoms were kept fixed. The atomic coordinates of the QM/MM-optimized structures are provided in the Supporting Information.

## Data availability

All data are available from the corresponding author (sudo@okayama-u.ac.jp) upon reasonable request.

## Supporting information

This article contains supporting information.

## Conflict of interest

The authors declare that they have no conflicts of interest with the contents of this article.

## Supporting information

Supporting Information

Atomic coordinates
