# Supplementary material for: Roles of basic amino acid residues in substrate binding and transport of the light-driven anion pump Synechocystis halorhodopsin (SyHR)
Source: J Biol Chem. 2025 Feb 19;301(4):108334. doi: 10.1016/j.jbc.2025.108334 (PMC11995866; doi:10.1016/j.jbc.2025.108334)
Supplement: Supporting Information [file mmc1.docx]

**Supporting Information**

**Roles of basic amino acid residues in substrate binding and transport of the light-driven anion pump *Synechocystis* halorhodopsin (SyHR)**

Masaki Nakama^1,#^, Tomoyasu Noji ^2,3,#^, Keiichi Kojima^1,4,#^, Susumu Yoshizawa^5^, Hiroshi Ishikita ^2,3^ & Yuki Sudo^1,4,*^

^1^Graduate School of Medicine, Dentistry and Pharmaceutical Sciences, Okayama University, Okayama 700-8530, Japan

^2^Department of Applied Chemistry, The University of Tokyo, 7-3-1 Hongo, Bunkyo-ku, Tokyo 113-8654, Japan

^3^Research Center for Advanced Science and Technology, The University of Tokyo, 4-6-1 Komaba, Meguro-ku, Tokyo 153-8904, Japan

^4^Faculty of Medicine, Dentistry and Pharmaceutical Sciences, Okayama University 700-8530, Japan

^5^Atmosphere and Ocean Research Institute, University of Tokyo, Chiba 277-8564, Japan

^#^These authors contributed equally to this work

*To whom correspondence should be addressed.

Yuki Sudo; Telephone: +81-86-251-7945, E-mail: [sudo@okayama-u.ac.jp](mailto:sudo@okayama-u.ac.jp)

**Table of Contents**

1. Supporting Figures S2-S6

2. Supporting Table S7

**Supporting Figures**

**
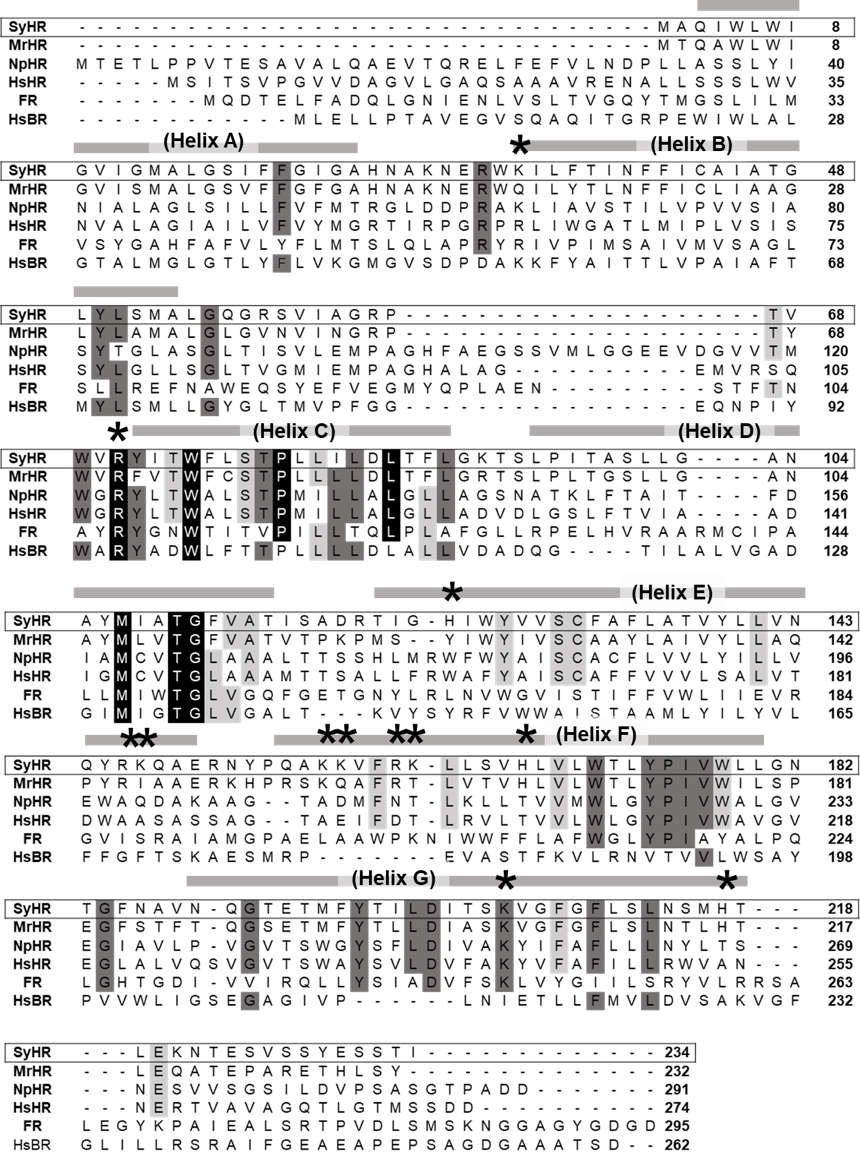
**

**Figure S1. Amino acid sequence alignment of SyHR and ClRs**

Multiple alignments of SyHR, typical ClRs, and bacteriorhodopsin was constructed using ClustalW. The accession numbers of the aligned sequences are as follows: *Synechocystis* halorhodopsin (SyHR), WP_009632765; *Mastigocladopsis repens* halorhodopsin (MrHR), WP_017314391; *Natronomonus pharaonis* halorhodopsin (NpHR), P15647; *Halobacterium salinarum* halorhodopsin (HsHR), P16102; *Fulvimarina pelagi* rhodopsin (FR), WP_007065598; *Halobacterium salinarum* bacteriorhodopsin (HsBR), CAP14056. Basic amino acid residues, marked by asterisks, indicate the mutation sites investigated in this study. Potential transmembrane regions are labeled as Helix A, B, C, D, E, F, and G.


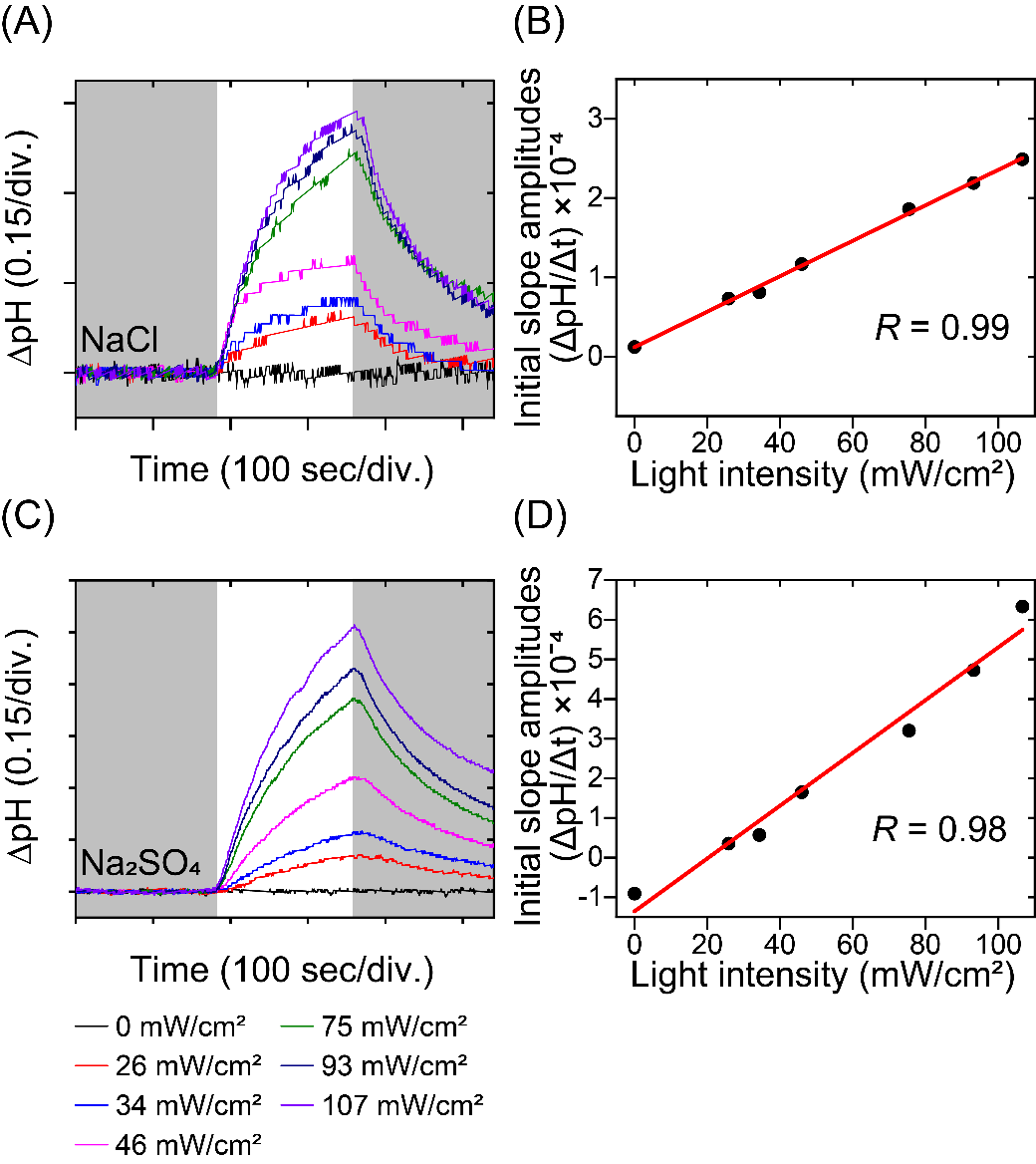


**Figure S2. Power dependence of the initial slope amplitudes of the light-induced pH changes by the wild-type SyHR**

(A, C) Light-induced extracellular pH changes of *E. coli* cells expressing the wild-type SyHR in solutions containing 300 mM NaCl (A) or 100 mM Na_2_SO_4_ (C) in the presence of CCCP. The cells were irradiated with yellow light (>420 nm) at varying light intensities (0-107 mW/cm^2^). (B, D) The initial slope amplitudes of the light-induced pH changes in solutions containing 300 mM NaCl (B) or 100 mM Na_2_SO_4_ (D) were plotted against the corresponding light intensities. The data were well-fitted by linear regression (red lines).


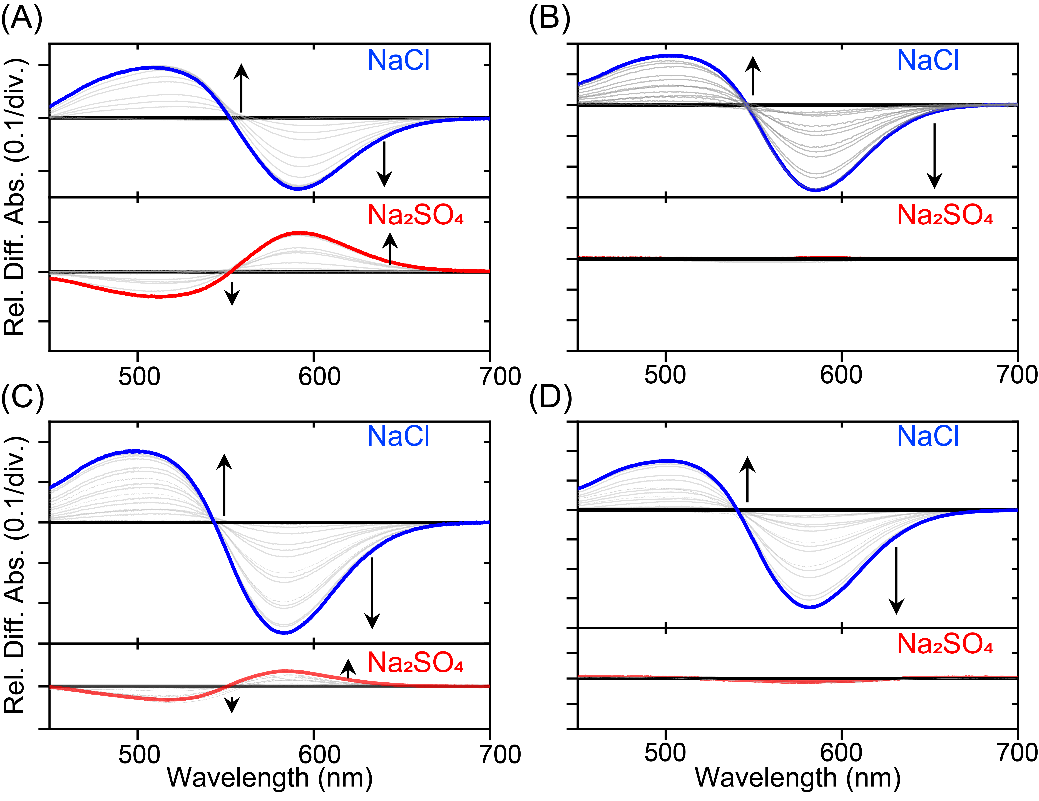


**Figure S3. Anion-induced spectral shifts of the wild-type and Arg71 mutants of SyHR**

Difference spectra of the wild-type (A) and the R71A (B), R71K (C), and R71Y (D) mutants of SyHR before and after addition of NaCl and Na_2_SO_4_.


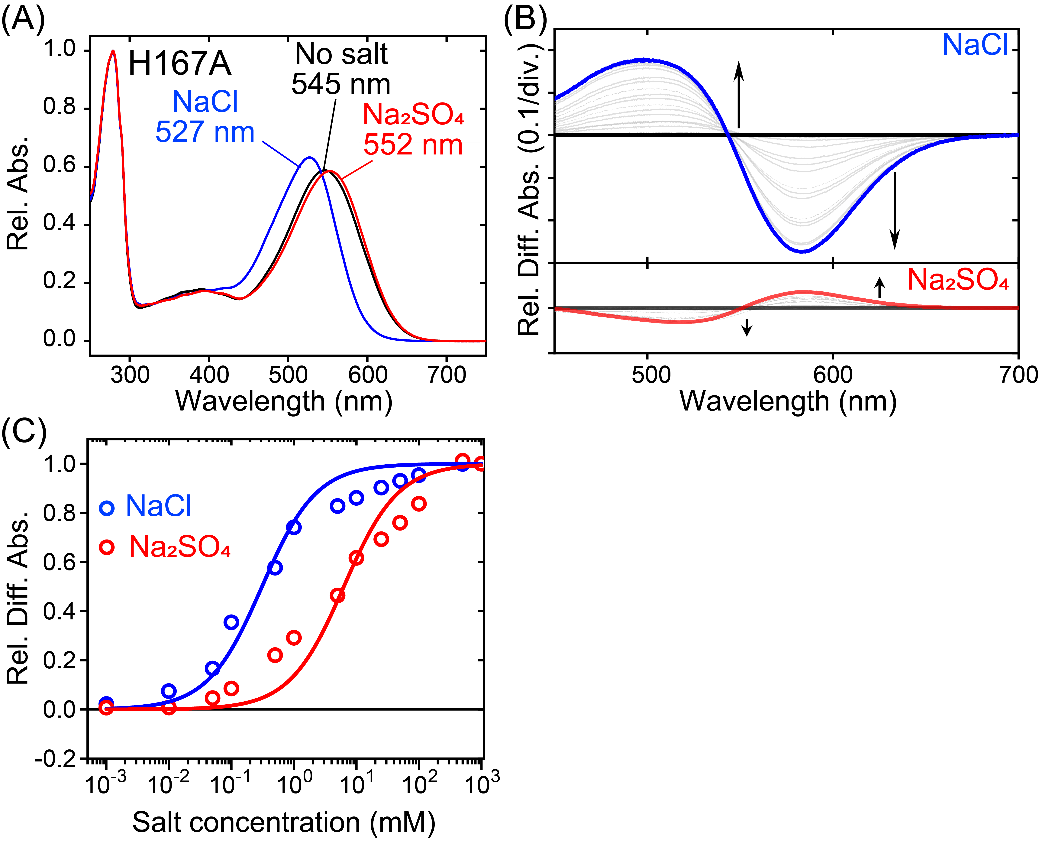


**Figure S4. Anion-induced spectral shifts of the H167A mutant of SyHR**

(A) Absorption spectra of the H167A mutant of SyHR in the presence or absence of salts (1M NaCl or 1 M Na_2_SO_4_). (B) Difference spectra of the H167A mutant of SyHR before and after the addition of NaCl and Na_2_SO_4_. (C) Absorption changes at the peak wavelengths in the difference spectra of the H167A mutant of SyHR are plotted against the salt concentrations. The data were fitted using a linearly combined Hill equation (solid lines) to estimate the affinity of the anions.


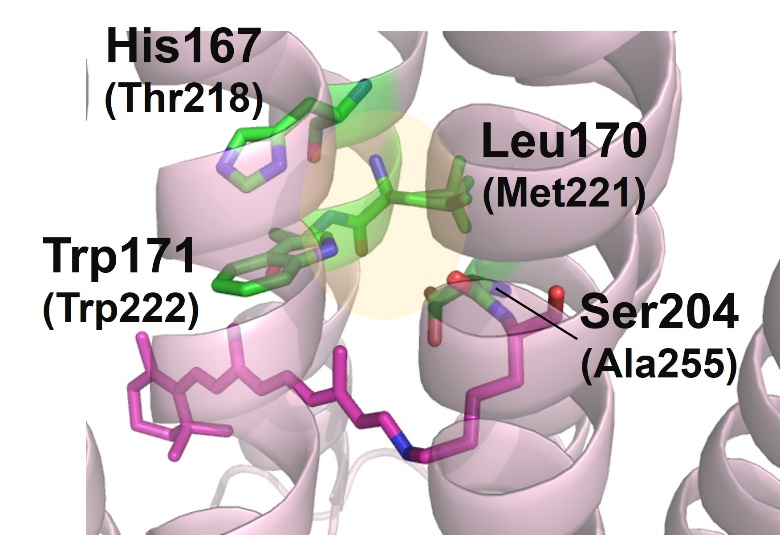


**Figure S5. Transient anion-binding site of SyHR**

The transient anion-binding site is constructed by the structure of SyHR (PDB: 7ZOU). Key residues of SyHR that form the binding site are indicated, with the corresponding residues of NpHR shown in parenthesis. The retinal and Lys205 are highlighted in magenta.

**Supporting Table**

**Table S1.** Fitting parameters of the Hill equation (Equation 2)

|  | Anion | *K_d1_* (mM) | *K_d2_* (mM) | *V_max1_* | *V_max2_* |
| --- | --- | --- | --- | --- | --- |
| Wild-type | Cl⁻ | 0.094 | 2.2 | 0.58 | 0.42 |
|  | SO_4_^2^⁻ | 1.2 | 82 | 0.45 | 0.55 |
|  |  |  |  |  |  |
| H71A | Cl⁻ | 0.052 | 22 | 0.35 | 0.65 |
|  | SO_4_^2^⁻ | — | — | — | — |
|  |  |  |  |  |  |
| H71K | Cl⁻ | 0.055 | 5.9 | 0.53 | 0.47 |
|  | SO_4_^2^⁻ | 0.47 | 93 | 0.58 | 0.42 |
|  |  |  |  |  |  |
| H71Y | Cl⁻ | 0.061 | 9.0 | 0.40 | 0.60 |
|  | SO_4_^2^⁻ | — | — | — | — |
